# A computation using mutually exclusive processing is sufficient to identify specific Hedgehog signaling components

**DOI:** 10.3389/fgene.2013.00284

**Published:** 2013-12-19

**Authors:** Spencer J. Spratt

**Affiliations:** ^1^Okinawa Institute of Science and Technology Graduate UniversityOkinawa, Japan; ^2^Department of Frontier Bioscience, Hosei UniversityKoganei, Tokyo, Japan

**Keywords:** genetics, computation, mutual exclusivity, specificity, methods, systems analysis

## Abstract

A system of more than one part can be deciphered by observing differences between the parts. A simple way to do this is by recording something absolute displaying a trait in one part and not in another: in other words, mutually exclusive computation. Conditional directed expression *in vivo* offers processing in more than one part of the system giving increased computation power for biological systems analysis. Here, I report the consideration of these aspects in the development of an *in vivo* screening assay that appears sufficient to identify components specific to a system.

## INTRODUCTION

Problem solving using computation is not only limited to silicon based systems. *Drosophila* genetics has been used for many years to help solve which genes interact and the biological processes they might be involved in (Martinez-Arias, 2008) and embodies the concept of a Universal Turing machine (Turing, 1936) whereby the computer can gain meaningful information from other machines. While serial processing is valid (Amdahl, 1967), parallel processing can offer increased computation power in terms of speed to help solve more complex problems within a practical time frame (Gustafson, 1988). The development of RNA interference (RNAi; Fire et al., 1998) together with directed expression using the Gal4/UAS system (Brand and Perrimon, 1993) has revolutionized the way we can examine putative gene loss of function in both a spatial and temporal manner *in vivo* (Dietzl et al., 2007; Perrimon et al., 2010). This reverse genetics approach offers easily accessible, highly parallel processing on the level of the number of genes that can be examined in a system and importantly on the level of the number of parts of the system they can be studied in. However, hitherto RNAi screening has been one dimensional: what I mean by this is that RNAi’s have been processed in only one part of the system lacking the power to determine inputs that might function specifically from those that function generally. This is an oversight leading to unnecessary downstream analysis of candidate genes that function generally and therefore inefficient use of resources when components specific to a system are sought.

**FIGURE 1:**
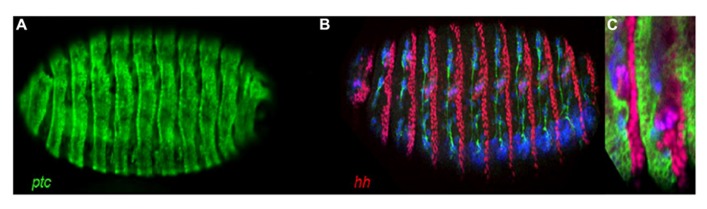
**Stage 16 *Drosophila* embryo’s showing an extensive and, for the most part, a mutually exclusive pattern of *ptc* and *hh* expression.** **(A)** *ptc-Gal4, UAS-GFP* showing *ptc* directed expression of GFP. **(B)** *hh-lacZ* Showing *hh* directed expression of LacZ (red). Note, here we can also see the post-mitotic neuronal marker Elav (blue) and the axon marker Neuroglian (green). **(C)** A region of a *ptc-Gal4, UAS-GFP; hh-lacZ* embryo showing for the most part a mutually exclusive pattern of *ptc* (green) and *hh* (red) expression.

A system of more than one part may exhibit general similarities between its parts but these will also exhibit specific differences. It is these differences when observed that allow us to gain meaningful information to describe the system. A simple way to do this is by observing something absolute displaying a trait in one part and not in another: in other words, mutually exclusive. Paracrine signaling systems such as Hedgehog (Hh) signaling provide a suitable model with which to explore the concept of mutually exclusive computation as they can be considered as consisting of two parts when they function in a paracrine manner: at the transcriptional level one part expresses the receptor, in this case *patched (ptc)* while its corresponding part expresses the ligand, in this case *hh* with their pattern for the most part mutually exclusive (**Figure 1**).

**FIGURE 2:**
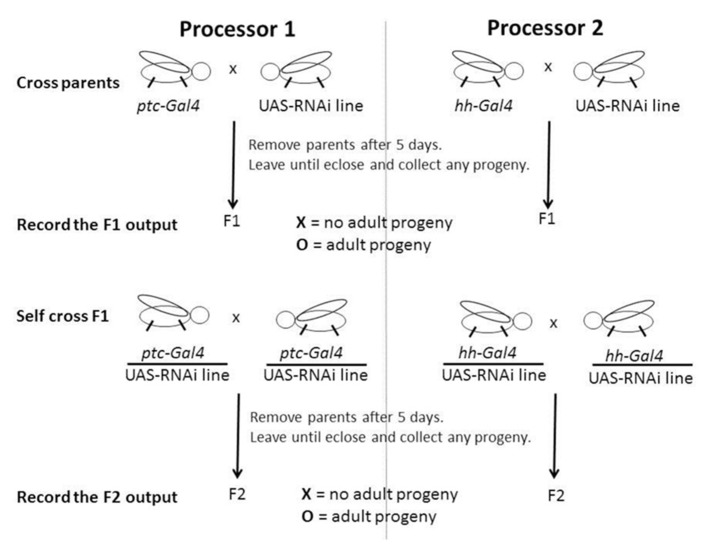
**Schematic of a computation using more than one *Drosophila* processors.** The directed expression commands and RNAi input generate the X or O output that is scored and recorded by asking IF F1 AND F2 = O put a O, OR IF NOT put an X. The two outputs for the *ptc* and *hh* directed parts of the computation generates a signature and might be O, O; X, X; X, O, or O, X which can be searched to identify genes that might function specifically with respect to the context in question. Please see main text for a detailed description of this method and **Table 1**.

Here, I demonstrate an application of mutually exclusive computation in the design of an *in vivo* RNAi screening assay examining the well characterized Hh signaling pathway as proof of the concept of a computation tool using *Drosophila* parallel processors to increase the power to identify candidate genes that might exhibit specificity (**Figure 2**). In addition to this methods efficiency its importance is emphasized by the need for drugs that can act in a specific context (Criscitiello et al., 2012). To highlight this with respect to Hh signaling and its involvement in cancer: several antagonists for Smoothened (Smo), the seven-pass trans-membrane positive transducer of the Hh signal, have been developed by drug companies. These have shown some success, for example in treatment of Basal Cell Carcinoma’s (Atwood et al., 2012). However, studies have revealed drug-resistant tumor variants that bypass Smo inhibition and in addition to the obvious concerns of targeting an important signaling pathway that is likely to play as yet unidentified roles in the adult, major issues include potential secondary developmental toxicities in children (Raju and Pham, 2012). Thus, there is a very clear need for tools that can identify context specific components of biological mechanisms to serve as potential drug targets.

## RESULTS AND DISCUSSION

### EXAMINATION OF THE F1 GENERATION

The secreted signaling molecule Hh and its signal transduction pathway is vital for multicellular organisms, playing multiple roles during development and in the adult including the control of stem cell fate (Ingham et al., 2011). Ptc is both a receptor for Hh and a direct transcriptional target of Hh signaling and is expressed at high levels in cells that receive the Hh signal (Chen and Struhl, 1996). Thus, we might expect *ptc* directed expression of RNAi lines for genes known to be Hh signaling components expressed in Hh signal receiving cells to have an effect, and for example in the case of *cubitus interruptus (ci)* we see lethality in the F1 generation, i.e., they are non-viable (**Table 1**) and I can record this F1 score using the symbol X. In turn, we might expect *ptc* directed expression of RNAi lines for genes not expressed in Hh signal receiving cells to not have an effect, and for example in the case of *hh* we see no lethality in the F1 generation and I can record its F1 score using the symbol O. These F1 scores can also be recorded as X and O attributable to an absence or presence of adult progeny, respectively (**Figure 2**). This is significant if we consider how a computer might record the F1 score. In the case of *ptc* directed *ptc* RNAi, while we see lethality in the F1 we also see adult progeny. This might be explained by the knockdown of *ptc* being compensated for, by the negative feedback when the pathway is activated increasing Ptc expression and serving to limit the extent to which the signal might be received by neighboring cells. This is interesting because depending on how the computer perceives the F1 will determine how the score is recorded. This question has implications if the assay is automated using a simple computer. Here, in the case of *ptc* RNAi I record the F1 score as an O because of the presence of F1 adults, but I acknowledge a more complex computer can record an X.

**Table 1:** A record of the results for a computation using *ptc* and *hh* directed expression of RNAi for genes, including those known to function in the Hh signaling pathway and its regulation.

Gene RNAi line	Directed expression				Signature
	*ptc*		*hh*	
	F1	F2	F1	F2
*ptc* (CG2411) 28795*	O^a^	X	O	O	X, O
*Cos2* (CG1708) 108914^#^	X^b^	–	O	O	X, O
*ci* (CG2125) 105620^#^	X^c^	–	O	O	X, O
*ci* (CG2125) 51479^#^	X^c^	–	O	O	X, O
*smo* (CG11561) 9542^#^	X^d^	–	X^e^	–	X, X
*smo* (CG11561) 27037*	X^f^	–	X^g^	–	X, X
*fu* (CG6551) 27662^#^	O^h^	O	O^i^	X^i^	O, X
*fu* (CG6551) 31495*	O^j^	O	O	O	O, O
*Su(fu)* (CG6054) 28559*	O	O	O	O	O, O
*hh* (CG4637) 1403^#^	O	O	X^k^	–	O, X
*hh* (CG4637) 43255^#^	O	O	X^l^	–	O, X
*disp* (CG2019) 0004^#^	O	O	O	O	O, O
*ttv* (CG10117) 4871^#^	X^m^	–	X^n^	–	X, X
*CkIa* (CG2028) 25786*	X^o^	X^o^	X^p^	–	X, X
*sgg* (CG2621) 31308*	O	O	O	O	O, O
*slmb* (CG3412) 107825^#^	X^q^	–	X^r^	–	X, X
*roc1a* (CG16982) 32399^#^	X^s^	–	X^t^	–	X, X
*lin19* (CG1877) 33406^#^	X^u^	–	X^v^	–	X, X
CG31522 37329^#^	O^w^	X	O	O	X, O
CG31522 106652^#^/CyO, GFP	X^x^	–	O	O	X, O
CG31522 106652^#^/CyO; *fu* (CG6551) 27662^#^/TM6b	O^y^	–	–	–	–
CG31522 106652^#^/CyO; *fu* (CG6551) 31495*/TM6b	O^z^	–	–	–	–
*N* (CG3936) 27229^#^	X^α^	–	X^β^	–	X, X
*Dl* (CG3619) 37288^#^	X^χ^	–	X^Ω^	–	X, X

Crosses were carried out in triplicate on different days. The commands and input generating the X or O output can be described as follows: processor 1 – *ptc-Gal4/ptc-Gal4* x RNAi line > F1, X = no adult progeny or O = adult progeny. F1 > F2, X = no adult progeny or O = adult progeny. Processor 2 – *hh-Gal4*/TM3, *Sb* × RNAi line > F1, X = no adult progeny (no non-Sb) or O = adult progeny (non-Sb). F1 (non-Sb) > F2, X = no adult progeny or O = adult progeny. If we consider each cross or processor separately, the result for the output of each can be gained by asking: IF F1 AND F2 are O put an O, OR IF F1 OR F2 is X, put an X. This can be simplified to IF F1 AND F2 = O, O, OR IF NOT, X. This output generates a signature and might be O, O; X, X; X, O; or O, X which can be searched to identify genes that might function specifically with respect to the system in question. *TRiP line Bloomington identifier. ^#^VDRC line identifier. ^a^Early instar lethal. Only female progeny but non-viable; ^b^Larval lethal; ^c^Pupal lethal. Some almost eclose; ^d^Pupal lethal; ^e^No non-Sb; ^f^Pupal lethal; ^g^No non-Sb; ^h^*Fused* wing; ^i^Non-sb but non-viable; ^j^*Fused* wing; ^k^No non-Sb; ^l^No non-Sb; ^m^Progeny die soon after eclosion; ^n^No non-Sb; ^o^Pupal lethal. A few escapers but non-viable; ^p^No non-Sb; ^q^Larval and pupal lethal; ^r^No non-Sb; ^s^Larval and pupal lethal; ^t^No non-Sb; ^u^Larval lethal; ^v^No non-Sb; ^w^Adult lethal. Dead in 3 days. No obvious morphological defect; ^x^Non-GFP non-CyO are first instar lethal. Do not move far. No obvious morphological defect; ^y^Mostly CyO progeny. A few were non-CyO non-TM6b; ^z^Mostly CyO progeny. A few were non-CyO non-TM6b; ^α^Pupal lethal; ^β^No non-Sb; ^χ^Pupal lethal; ^Ω^No non-Sb

### EXAMINATION OF THE F2 GENERATION

Hh signaling mediates homeostasis in several adult niches. In the *Drosophila* gonads Hh signals directly to drive the proliferation of somatic stem cells in both the adult ovary (Forbes et al., 1996; Zhang and Kalderon, 2000) and testis development (Michel et al., 2012; Amoyel et al., 2013). The loss of Hh signaling here can cause male sterility, i.e., no F2 generation (Christian Bokel, personal communication). These somatic cells go on to ensheath the germline cells and Hh signaling may play an indirect role in their development. Recently, a study including the use of directed RNAi shows an indirect role for Hh signaling to the supporting escort cells maintaining the normal pool of germline stem cells (GSCs) in the adult ovarian niche (Rojas-Rios et al., 2012). While, RNAi lines for all genes might not appear to be effective in the female germline (Perrimon et al., 2010), an explanation for this might include timing of RNAi expression and the extent of maternal contribution for a corresponding mRNA. Still, this concern over potency does not include the somatic cells. Thus, I decided it to be appropriate to include in the assay an examination of the F2 generation (**Figure 2**). Interestingly, for *ptc* directed *ptc* RNAi, there were no F2 progeny and I record this F2 score as X (**Table 1**). Thus, according to the assay I describe, in the case of *ptc* directed *ptc* RNAi while the F1 score taken alone might be considered as a false negative result, the F2 score produces an overall score of X and like *ptc* directed *ci* RNAi can be considered as non-viable and a positive result. Here, I am describing an assay capable of identifying Hh signaling components simply using directed RNAi *in vivo*. I do this in a way that can be described as examining viability, firstly by including an indirect examination of F1 lethality or if not lethal by including a second indirect examination of the F1’s ability to reproduce. For both counts this is done by examining whether there is an absence or presence of adult progeny in the F1 and F2 respectively, and the corresponding result is recorded using the two symbols X and O. It is beyond this studies scope to characterize the changes caused by the RNAi in detail. In several instances using this assay where I have seen F1 adults but no F2 progeny (unpublished observation), F2 lethality can be ruled out because the F1 are heterozygous for both driver and RNAi. This means we expect to see progeny including combinations lacking one or both these elements and these can be considered essentially normal. Non-viable F1 might result from an effect on the germline indirectly through somatic cell regulation in the reproductive organs but we should also consider that, Hh signaling functions again and again throughout development and it is interesting that Ptc appears to be expressed at high levels in the adult nervous systems (Gazi et al., 2009). However, studies are scarce concerning a specific role in later nervous system development through conditional loss of function studies. Still, it is worth noting that later nervous system defects, for example nerve synapse defects, whether at a neuromuscular junction or a nerve to nerve synapse can cause the neuromusculature or controlling nervous system to function abnormally leading to defects including progressive loss of locomotion, causing indirect larval, pupal, or adult lethality (Chan et al., 2003; Gazi et al., 2009) and behavioral defects, for example courtship abnormalities (Finley et al., 1997). It has been known for some time that in certain instances, for example loss of the gene *dissatisfaction (dsf)*, flies can be non-viable, i.e., the F1 are unfit to reproduce, due to nervous system defects including Bouton defects at specific nerve muscle synapses (Finley et al., 1997). Therefore, it is reasonable to assume that this assay, in addition to the possibility of identifying genes that might function later in the development of the sex organs and homeostasis of the germline in this context, might also offer the potential to identify genes affecting the F1’s ability to reproduce indirectly through the control of other aspects of biology, and potential defects of this type could still be broadly defined as a viability defect. Furthermore, Hh signaling is known to function in additional later stem cell niches, for example in the larval lymph gland maintaining the blood cell precursors (Mandal et al., 2007; Mondal et al., 2011) and in the adult hindgut regulating the differentiation of stem cells (Takashima et al., 2008). Therefore, it will be interesting to disrupt Hh signaling in these contexts and examine the output in terms of viability and also longevity of these animals since a simple adjustment to this type of assay, could examine the life span of the F1 adults.

### DIRECTED EXPRESSION USING TWO INSTRUCTIONS THAT SHARE A MUTUALLY EXCLUSIVE RELATIONSHIP

The use of *ptc* directed expression alone for the assay lacks the power to differentiate those components that appear to be specific to Hh signaling, i.e., normally appear to function only in Hh signal receiving cells or cells with the potential to receive the Hh signal, for example *ptc,* from those components we know function in other systems, for example Casein kinase Ia (CkIa; Price, 2006). In *Drosophila*, *ptc* and *hh* are expressed extensively, and for the most part in a mutually exclusive pattern during embryonic development (**Figure 1**). Hh signaling is known to be used again and again throughout development and in the adult of multicellular organisms (Ingham et al., 2011) generally using paracrine and juxtacrine mechanisms. Thus, if we consider all cells expressing *ptc* and all cells expressing *hh* together, we can say they overlap with the expression patterns of components of multiple biological systems throughout development and in the adult. Therefore, to gain power and differentiate genes that may or may not function specifically in paracrine Hh signaling, I performed dual *ptc* and *hh* directed RNAi processing (**Figure 2**; **Table 1**).

Dual processing each RNAi now gives four possible scenarios. I reasoned that for those genes that might function specifically in Hh signal receiving cells, parallel processing their RNAi using *ptc* and *hh* directed expression might result in an X and an O score respectively if a candidate genes function is mutually exclusive thus fitting the paracrine model for Hh signaling: I term this an X, O signature. The more general machinery known to function in multiple systems might result in an X and an X score respectively: I term this an X, X signature. In addition, we might expect an X, X signature for genes specific to other vital systems that function in contexts other than Hh signaling. The machinery not vital might result in an O and an O score respectively: I term this an O, O signature. In turn, for genes that might function specifically in Hh sending cells the result might be an O and an X score respectively: I term this an O, X signature. While X, X and O, O signatures can be considered as negative results, the X, O and the vice versa O, X signatures can be considered interesting because they indicate specificity and depending on the type of genes sought by the user both could be regarded as positive results in this type of assay. However, foremost, I designed this assay to search for genes that might function specifically in Hh signal receiving cells returning an X, O signature.

I found for several genes that we might expect to fit the patterns described above indeed it was the case. For *ptc* directed *ptc, Costal2 (Cos2),* and *ci* RNAi the result is non-viable while *hh* directed expression is viable: an X, O signature (**Table 1**). For *CKI, slmb,* and *roc1a*, genes known to function in other systems the result is non-viable in both parts: an X, X signature. As we might expect for *ptc* and *hh* directed *hh* RNAi the result is viable and non-viable respectively: an O, X signature. In addition to the importance of this control giving validity to the assay, this result merits comment as it indicates that this assay might also offer an opportunity to search for genes that might function specifically in Hh signal sending cells with respect to Hh signaling. Also, if we consider that we do not know a single more precise Hh signaling context specific gene to use to direct expression, i.e., one that might only be used in a sub-set of contexts where Hh signaling might be used, the potential identification being part of the impetus behind this study: the use of two genes to direct expression or instructions that share a mutually exclusive relationship importantly appears to give us sufficient power to identify those candidates that exhibit specificity. However, I do accept that this assay as specifically described here would be insufficient to determine potentially specific autocrine functioning components of Hh signaling. Furthermore, while the assay appears to provide the power to identify those genes that are generally accepted to function specifically in Hh signaling, the assay might identify other genes that exhibit a relationship with Hh signaling: for example any targets of the pathway that might be specific or candidates that while not a component of the pathway might function specifically in the same cells as the system being searched.

### IDENTIFICATION OF CG31522

The proof of the concept I report here raises an interesting question: can this type of assay show beyond a reasonable doubt that components identified function specifically in a system described by the instructions? With this in mind, I would like to report the X, O output or signature generated for two RNAi lines (**Table 1**) corresponding to CG31522 a putative Elongase of very long chain fatty acids (ELOVL)^1^. This putative protein encoding gene first came to my attention as it appeared to show reduced expression in a global gene expression study of late stage *smo*^D16^ (van den Heuvel and Ingham, 1996; Chen and Struhl, 1998) embryo’s at the transcript level (unpublished observation). Interestingly, the X, O signature is identical to those generated for *ptc, Cos2,* and *ci* and therefore can be considered as genetic evidence that CG31522 might share a relationship with Hh signaling. In addition, both *fu* RNAi lines used in this study appear to suppress the late embryonic lethality seen with the *ptc* directed CG31522 106652 RNAi line generating adult progeny (**Table 1**). This important further genetic evidence indicates that the known Hh signaling component *fu* is epistatic to CG31522 and gives strong support to the notion that CG31522 might be a novel Hh signaling pathway component. Interestingly, recent studies recorded on FlyBase^2^ using coaffinity purification coupled to mass spectrometry provides evidence that the putative protein encoded by CG31522 interacts with its homolog, the protein encoded by the adjacent gene CG31523 (Guruharsha et al., 2011). With respect to Hh signaling: this data, together with previous data generated using yeast-two-hybrid (Y2H) based technologies and searchable using InterologFinder^3^ (Wiles et al., 2010), indicates that CG31522 or possibly via CG31523 interacts indirectly with the Hh signaling components Slmb and Ci (Giot et al., 2003; Formstecher et al., 2005; Guruharsha et al., 2011). One of these proteins shown to interact with CG31522 is Protein Phosphatase 2A (PP2A), a phosphatase shown to specifically dephosphorylate Smo restricting signaling by high concentrations of Hh (Su et al., 2011). Interestingly, another tandemly arranged duplicated gene (Quijano et al., 2008) and putative ELOVL *bond* disrupts *Drosophila* spermatocyte division (Szafer-Glusman et al., 2008). However, it is studies in vertebrates that provide the majority of information concerning the role for this type of gene. Importantly, a study identified a putative human ortholog ELOVL7, as overexpressed and involved in prostate cancer growth and cell viability (Tamura et al., 2009). What’s more, previously another putative ortholog ELOVL4, known for its role in degenerative diseases of the macula tissue, a central part of the eye retina, is able to rescue the neo-natal lethality seen in a mouse model of this type of disease (McMahon et al., 2011). Lastly, a report in mice is of note because it describes another ortholog, ELOVL3 expressed in skin cells with the implication that these cells might be stem cells (Westerberg et al., 2004). Taken together, I believe this information underlines that this type of gene should be prioritized for further study concerning their function and potential involvement in Hh signaling.

### FURTHER CONSIDERATIONS USING THIS TYPE OF ASSAY

Using this assay, while for several Hh pathway components not known to function in another system the results return a positive X, O signature and thus fit our model, for some genes generally accepted to be of this type, this is not the case, for example *smo,* and *fu* and *Suppressor of fused [Su(fu)]* return results that appear to be false negatives. In some instances, discrepancies of this type might be explained due to off-target effects (OTEs; Mohr and Perrimon, 2012), however, it is unlikely to be a sufficient explanation in all instances and the reader should be aware of other possible explanations that serve to highlight the limitations using this assay as presented here and what appears to be the high chance of false negatives.

A negative X, X signature as in the case of *smo* might be explained by an autocrine function but this is less likely with little evidence for Hh signaling function in this manner in flies and supported by those components resulting in positive X, O signatures. Another possibility that could generate an apparent false negative of the type X, X is if a components function is shared with another system. This might be in a system whose expression overlaps with Hh signaling or whose relationship is more closely entwined, for example in an interacting pathway that shares some cross-talk with Hh signaling (Takebe et al., 2011). However, there is little evidence for Smo function in this manner in *Drosophila* so while this apparent false negative result could be interesting it requires further investigation to confirm whether this is the case or not.

In the case of *fu*, the characteristic fusion of the third and fourth wing veins (Preat, 1992) was evident – highlighting the potential for identification if the assay is adjusted and the computer records a different output – but depending on the RNAi line examined, resulted in an O, X or O, O signature. The score of X in the case of *hh* directed expression, in addition to the possibility of OTEs it is worth considering that *fu* is known to function regulating the BMP receptor Thickveins (Tkv) in ovary GSC derived cystoblasts (CB; Xia et al., 2010). Interestingly, Hh appears to be expressed in the GSC producing primordial germ cells (PGCs) during late larval development (Sato et al., 2010). Perhaps *fu* RNAi might be present at sufficient levels to disrupt CB differentiation and normal oogenesis. However, it is surprising that the *ptc* directed *fu* RNAi result is viable considering its role in somatic cell differentiation in egg chamber formation (Besse et al., 2002). Thus while in the first instance perhaps a plausible explanation might be due to function in another system, a possibility for the latter might be due to compensation by the system: the RNAi knockdown might be insufficient and compensated by a suppressor, in this example we have a candidate in *Su(fu)*.

The negative O, O signature type for *Su(fu)* might be expected since *Drosophila* lacking *Su(fu)* are known to develop into viable and fertile adults (Preat, 1992). Thus, while this apparent false negative might be explicable it none the less highlights a further limitation using this assay in that other suppressors will probably be missed and if sought the user should consider an adjustment of this assay perhaps similar to that used in the screen to identify *Su(fu)*. Essentially, Preat’s assay is designed to identify interacting genes and can therefore be considered as a forerunner for assays with the capability to search for multiple genes involved in a function, perhaps functioning in a complex, in other words what might be regarded as a functional set. This has some similarity with the potential for the type of assay described here and is worth some thought. Interestingly, an OTE might result in a second target that by chance suppresses the intended target. While on the one hand this appears to be a hindrance in our assay, at least in the potential for the return of inaccurate results without full awareness of OTEs that might occur, on the other hand serves to highlight the potential for this type of assay to interrogate more than one component that will probably have important applications. With the idea for multiple targets in mind, we can see potential for this type of method perhaps not only confined to reverse genetics but also with a forward genetics aspect if, in addition to scope for the development of targeting combinations of genes, we might also include randomness in the generation of the sequences used.

I think it is true for most, if not all assays that we might expect a number of false negatives but this is can be considered outweighed by an assays ability to identify true positives. Still, to give further support to the accuracy of this assay I processed RNAi lines corresponding to the genes *Notch(N)* and *Delta (Dl)*, the receptor and one of its ligands involved in another cell signaling mechanism used again and again throughout development and in the adult (Artavanis-Tsakonas et al., 1995), as we might expect both returned the negative result of X, X (**Figure 1**). Furthermore, from the examination of RNAi lines corresponding to around 100 genes, the observation of an X, O signature was rare (data not shown) indicating that false positives are likely not to be problematic using this assay searching for the X, O output. However, I did see the O, X signature several times indicating this assay as it stands might not be well suited to the search for genes showing specific function in Hh sending cells. Still, as mentioned above, foremost I designed this assay to search for genes that might function specifically in Hh signal receiving cells returning an X, O signature. Therefore, this concern over an overly sensitive Processor 2 (**Figure 2**), i.e., a return of an X score for RNAi lines processed using this *hh-Gal4* line for *hh* directed expression, should be considered if genes that might function specifically in the Hh signal sending cells are sought. However, importantly this apparent sensitivity is likely to be beneficial in the accuracy of the search for genes that return an X, O result, increasing the proportion of true positives identified reducing the resources spent upon downstream analysis of potential false positives.

Also, I think it is worth briefly mentioning here, numerous instances of screening assays use lethality as an identifier, for example Nusslien-Volhard and Weischaus in their landmark paper (Nusslein-Volhard and Weischaus, 1980) first identify mutants in this way but then look on a second level to identify those involved in the early biological phenomenon of segmentation. The assay I report here, for example in the case of *ci*, appears to have the ability to show a genes involvement in a biological phenomenon, in this case a specific function in the context of Hh signaling, from examining lethality alone, or to be more exact from showing gene knockdown in two related parts of a system to be non-viable and viable, respectively.

## CONCLUSION

In conclusion, using Boolean operators (Boole, 1848): i.e., using AND, OR, and NOT to count unambiguous output of O or X, from two related yet mutually exclusive instruction streams or commands and inputs: i.e., examination of F1 and F2 generations from *ptc* and *hh* directed processing of putative RNAi lines of genes, I demonstrate an *in vivo* assay with the ability to identify genes that appear to function specifically in Hh signaling. This assay can be considered as novel because it is the first time that I am aware of, that a screening assay has been demonstrated to show a genes relationship with a specific biological phenomenon from examining non-viability (including lethality) and viability in respective parts of a system consisting of at least two related yet mutually exclusive parts. Therefore, this work demonstrates for the first time, the potential power of directed expression using mutually exclusive processing (in this case of putative RNAi lines of genes). Taken together, if we consider that *Drosophila* are self-assembling and renewable so that a one-time use from command and RNAi input to output is not limiting and examination of viability can be easily automated using a simple computer, for example using motion sensors, then in effect I am describing a prototype for a computer that utilizes *Drosophila* processors to compute the relationship between systems and their components that might play context specific functions vital for life.

**FIGURE 3:**
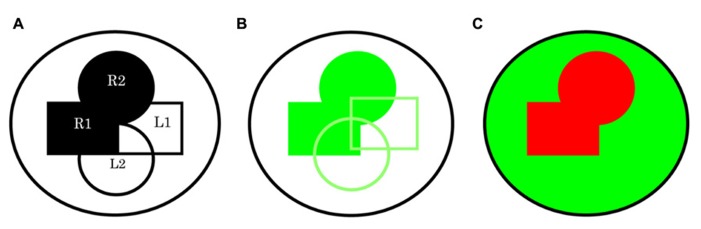
**A schematic representing directed expression using multiple drivers without and with the use of a repressor system. (A)** In this simple depiction of two paracrine signaling pathways within a whole system the receptors R1 and R2, and their respective ligands L1 and L2 can be thought of within a few cells in one space and time or the sum of multiple events. **(B)** The possibility for shared expression brings us to the realization that using all drivers R1, R2, L1, and L2 to direct expression of RNAi for a common component even if specific to both pathway receiving cells, might return a score of X in all instances. An X, X signature is likely if R1 plus R2 versus L1 plus L2 drivers are used in these two combinations. **(C)** However, this limitation can be circumvented if we use a repressor system: for example, a global driver such as *tubulin-Gal4* together with R1-*Gal80*, R2-*Gal80*. If a combination of R1-*Gal4* and R2-*Gal4* drivers is now used in parallel an X, O signature is possible.

I think it is important to realize that while the assay I demonstrate here may have its practical limitations, still the concept of mutually exclusive computation in the study of biology and probably not only limited to this science, has important implications. There will be potential for optimization using new technology as it becomes available. A possibility would be to make use of the Gal4 repressor Gal80 (Ma and Ptashne, 1987; Lee and Luo, 1999): for example, *ptc-Gal4* and in parallel a global driver such as *tubulin-Gal4* plus *ptc-Gal80* or instead the use of the ligand driver might be preferable still. This should ensure mutually exclusive expression that might be preferable commands to direct inputs for this type of study because it could offer an opportunity to solve the current limitation in the assays inability to identify specific autocrine components. Interestingly, use of this repressor technology combining multiple drivers might be a way to address questions concerning potential function in interacting pathways as it appears possible to generate mutually exclusive driver expression that might not be possible using multiple individual Gal4 drivers if there is a chance of expression overlap (**Figure 3**). Furthermore, it is interesting that this type of assay has the potential to identify additional relationships: for example, a Universal Turing machines (Turing, 1936) next computation is a product of its last, therefore this computers next command could be directed in this way: i.e., if a computation has just identified a gene *x* with an X, O signature the next command might be *x-Gal4* and in parallel *tubulin-Gal4*, *x-Gal80* and so on. It is clear, we are entering into very exciting times with *in vivo* systems analysis.

To highlight the important implications of this type of assay further: studying Hh signaling later in development has been difficult because its unconditional loss is early embryonic lethal. The question of how Hh signaling might function in different contexts to bring about different cell fates has only been touched upon and still eludes us for the most part. Hh signaling is involved in late aspects of development and disruption, at least in the case of Hh signaling in the gonads can cause the F1 to be non-viable. Thus, if we can accept that RNAi is potent in somatic cells then an effect on late aspects of development including, but perhaps not only limited to, later germline development, will be picked up using this assay, then it allows us to make the conclusion that: This assay might offer a simple and powerful way to examine this question concerning finding genes involved in later Hh signaling function. Answers to this question appear paramount in the consideration for targeting Hh signaling in childhood cancers in an effort to bypass potential secondary developmental toxicities (Raju and Pham, 2012). The assay could be used for a genome-wide screen or as a secondary assay, for example to analyze the ever increasingly complex gene expression data (Carninci, 2010).

Also, here I provide the first compelling genetic evidence that the putative protein encoding gene CG31522 shares a relationship with Hh signaling. This observation is significant considering the information provided from vertebrate studies concerning the function of its putative orthologs including an involvement in cancer cell growth. Together with the aforementioned protein interaction studies, my conclusion is that these genes should be given high priority for further study to examine their function more closely and any relationships they might share with Hh signaling and stem cell fate.

To end with: it’s worth considering how we define cell signaling mechanisms, including Hh and how this is changing as our understanding concerning their context specific function increases (Robbins et al., 2012). To be Hh, or not to be Hh, that is the question?

## MATERIALS AND METHODS

### FLIES AND CROSSES

All flies can be obtained from the Bloomington stock center or VDRC. The cross scheme and its scoring method is as described in **Table 1**: six virgin females of either *ptc-Gal4*/*ptc-Gal4* or *hh-Gal4*/TM3, *Sb* were crossed with three males of each RNAi line used, and left for 5 days before removing them. Flies were raised at 25°C on standard media and crosses were left at 29°C.

### IMMUNOHISTOCHEMISTRY AND MICROSCOPY

Embryos were dechorionated in bleach, washed in PBS with 0.1% Triton X-100, and then fixed in 1:1 PBS, 4% paraformaldehyde:heptane for 20 min. After washing with heptane, embryo’s were vortexed vigorously for 30 s in 1:1 heptane:methanol. Devitellinized embryos were washed three times in methanol and then probed using antibodies according to standard procedures before being examined using a Zeiss LSM510 Meta Confocal microscope. Primary antibodies were obtained from the Developmental Studies Hybridoma Bank (DSHB) except for Rabbit anti-LacZ (Stratech Scientific). Fluorescently labeled secondary antibodies raised in donkey were obtained from Jackson Immunoresearch Laboratories. All antibodies were used at their recommended concentrations.

## Conflict of Interest Statement

The author declares that the research was conducted in the absence of any commercial or financial relationships that could be construed as a potential conflict of interest.

## AUTHOR CONTRIBUTIONS

Conceived and designed the experiments: Spencer J. Spratt. Performed the experiments: Spencer J. Spratt. Analyzed the data: Spencer J. Spratt. Contributed the program for analysis: Spencer J. Spratt. All other reagents and materials are publicly available. Wrote this paper: Spencer J. Spratt.

## Footnotes

1 www.flybase.org/reports/FBgn0051522

2 flybase.org

3 http://interologfinder.org/simpleIfWS
